# A taste for beauty: On the expected taste, hardness, texture, and temperature of geometric shapes

**DOI:** 10.1177/20416695221120948

**Published:** 2022-09-19

**Authors:** Georgiana Juravle, Emilia-Liliana Olari, Charles Spence

**Affiliations:** 112930 Alexandru Ioan Cuza University, Iasi, Romania; Oxford University, UK

**Keywords:** geometric shape, symmetry, Platonic solids, taste, temperature, texture

## Abstract

Rounded shapes, which have been shown to enhance sweetness, were compared to the perfectly symmetrical Platonic solids. In a first online experiment, participants were presented with a rotating three-dimensional geometric shape (a sphere, the five Platonic solids, and three irregular angular/rounded/naturalistic controls), and indicated their liking for the shape, as well as its perceived hardness, and its expected temperature. The sphere was liked best, followed by the Platonic solids. The sphere was also evaluated as softest, and received the warmest temperature ratings. By contrast, the Platonic solids were rated as harder and significantly colder than the sphere. Experiment 2 investigated whether the liked shapes were also evaluated as looking tastier. Ratings of expected tastiness and the appearance of five shapes selected based on high liking scores and fitted with edible and inedible visual textures were recorded. The sphere was rated as looking tastiest, with edible-textured rounded shapes resulting in significantly tastier ratings. Experiment 3 assessed the taste corresponding to each shape. A sweet and umami preference for rounded shapes was documented, with sour and bitter typically matched to angular shapes. Importantly, the Platonic solids were associated with several tastes. These findings are explained in terms of current theories of crossmodal correspondences, while considering how temperature and texture can be used to modulate expected liking.

## Introduction

As one of the main criteria used in the visual identification of the objects in our environment, judgments of shape are implicated in almost every aspect of our daily lives. Furthermore, people typically prefer rounded versions/contours of objects, which are described as looking more beautiful. This preference for rounded shapes is not unique to humans, as it has also been documented in other species (Munar et al., 2015), and may be linked to the roundness that is common in nature (see Lee, 2018). Although the origin of the preference is not entirely clear, the phenomenon is well-known amongst both artists and scientists (Silvia & Barona, 2009). The enhanced liking of rounded shapes has been documented in various industries and fields of research, such as the design of typeface (Kastl & Child, 1968), logos (Jiang et al., 2016), cars (Leder & Carbon, 2005), toys (Jadva et al., 2010), as well as a range of other everyday objects (Bar & Neta, 2006; Ghoshal et al., 2015). With respect to taste, it has been demonstrated that people tend to evaluate food presented on rounded plates as tasting sweeter, whereas the basic tastes of bitter, sour, and salty are typically associated with angular shapes (Fairhurst et al., 2015; Stewart & Goss, 2013; Velasco et al., 2015; though see also Piqueras-Fiszman et al., 2012; for a study where the shape of the plate failed to modulate people's appreciation of the food that was placed on it). The sweet-round correspondence extends beyond the realm of taste perception: Rounded furniture and even environments may also prime sweetness (Spence, 2020b). Moreover, people have been reported to drink alcoholic beverages more rapidly from curved glasses and evaluate the taste of their beer as being more fruity and intense, when their glass is rounded (Attwood et al., 2012; Mirabito et al., 2017).

**Figure 1.**
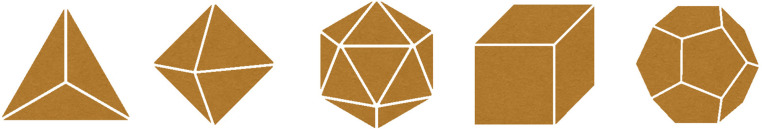
Examples of the five Platonic solids, from left to right: tetrahedron, octahedron, icosahedron, cube or hexahedron, and dodecahedron.

In the context of rounded geometric shapes, the sphere appears to be one of the most common natural forms and is thought to symbolize the purest expression of form and harmony (Popko, 2021). On the other hand, the Platonic solids are famous for their beauty and complexity from very early times (Lloyd, 2012), having been considered in various fields of the arts, religion, philosophy, and design, as well as in the sciences, in mathematics, chemistry, and architecture (Emmer, 1982; Sala & Cappellato, 2019). The Platonic solids take their name from the philosopher Plato, who first described them in his dialogue *Timaeus*. There are five Platonic solids (tetrahedron, octahedron, icosahedron, cube or hexahedron, and dodecahedron), see Figure 1. These are regular convex polyhedra with all faces formed of regular congruent polygons meeting at each vertex, such that each of the Platonic solids is *perfectly symmetrical*—no matter which direction it is turned/rotated. Importantly, the regular geometric shapes of the Platonic solids can be encountered in diverse natural aspects of everyday life, both animate (e.g., DNA of simplest life forms, such as marine algae, or viruses) and inanimate (e.g., in the structure of crystals). Plato considered them to be the building blocks of the natural world/universe, hinting, in their perfect symmetry, as having the potential to derive beauty from geometrical symmetry (Wilczek, 2015).

Geometrical symmetry allows for transformations in any part of an object, while the object in its entirety remains unchanged, a property also described as *invariance*. To exemplify invariance, take the case of a sphere rotating around its center for any given angle: With each rotation, the shape of the sphere will be visually reproduced, thus demonstrating a continuous group of symmetry transformations. Turoman and her colleagues demonstrated the symmetry group played a crucial role in shape–taste crossmodal correspondences (Turoman et al., 2018). Their study opposed shapes with different symmetry groups (symmetry along a single axis, left–right, reflectional, vs. symmetry along multiple axes, reflectional and rotational) to show that objects with multiple axes of symmetry are perceived as most pleasant and tend to be associated with a sweet taste. Results such as these provide support for enhanced liking and perceived tastiness/sweetness in regular geometric shapes used crossmodally in shape–taste correspondences research, as opposed to the typical correspondence found between asymmetrical shapes and sour taste (Deroy & Valentin, 2011). In this context, the question that arises regards whether liking or beauty and/or tastiness, with respect to taste perception, results from *geometric regularity* or *symmetry* (as given by a geometric shape being regular or not, irrespective of the classical angular vs. rounded shape divide), or rather, the *rounded shape effect* will always take precedence as far as liking and aesthetic judgements are concerned. We approach this question by assessing people's liking of rounded versus Platonic shapes in a first study. We then take the Platonic solids that were most liked together with the sphere in a second study, where we test them crossmodally, by pairing the various geometric shapes with edible and inedible textures. Our third study assesses the basic taste that corresponds to each of the nine geometric shapes used in the three experiments reported here.

As such, in Experiment 1, the participants were presented with a selection of geometric shapes including the five Platonic solids and a sphere, two irregular geometric objects custom-built such that they appeared as having an irregular angular shape and an irregular rounded shape (these two shapes were constructed to afford a classical comparison with Kiki-/Bouba-like shapes, see, e.g., Spence & Deroy, 2014), and the last irregular control object modeled from a naturalistic rock. Our approach is novel because we provide participants with rotating three-dimensional (3D) geometric shapes and we consequently assure a dynamic assessment of symmetry. Experiment 1 thus allows for a proper assessment of rotational symmetry, with the geometric shapes being in addition characterized sensorially with regards to hardness and temperature. We hypothesize that judgments of liking for the geometric shapes will be significantly impacted by their geometric regularity or symmetry, as well as their shape.

## Experiment 1

### Methods

#### Participants

An a priori power calculation with an *α* = .05 and 1−*β* = .80 indicated that a sample size of 28 participants would be necessary to detect an *f* = .25 effect in the data (G*Power 3.1; Erdfelder et al., 2009; Faul et al., 2007), should one be present (Cohen, 1988). In fact, a total of 41 participants took part in Experiment 1; six of whom were excluded from the final sample following an outlier exclusion in the reaction time (RT) data, based on the *z*-score > 3 rule (Juravle & Spence, 2015; Pukelsheim, 1994). The remaining sample (*N* = 35, 23 female) had a mean age of 23 years (*SD* = 7 years, age range: 18–57 years). Participants were recruited from socializing apps (e.g., WhatsApp, Instagram, and Facebook) and gave their informed consent to take part in the study. The study was approved by the Ethics Board of the Alexandru Ioan Cuza University of Iasi, Romania (no. 2325/24.05.2021). This study conforms to the Declaration of Helsinki (World Medical Association, 2013).

#### Materials and Apparatus

**Figure 2.**
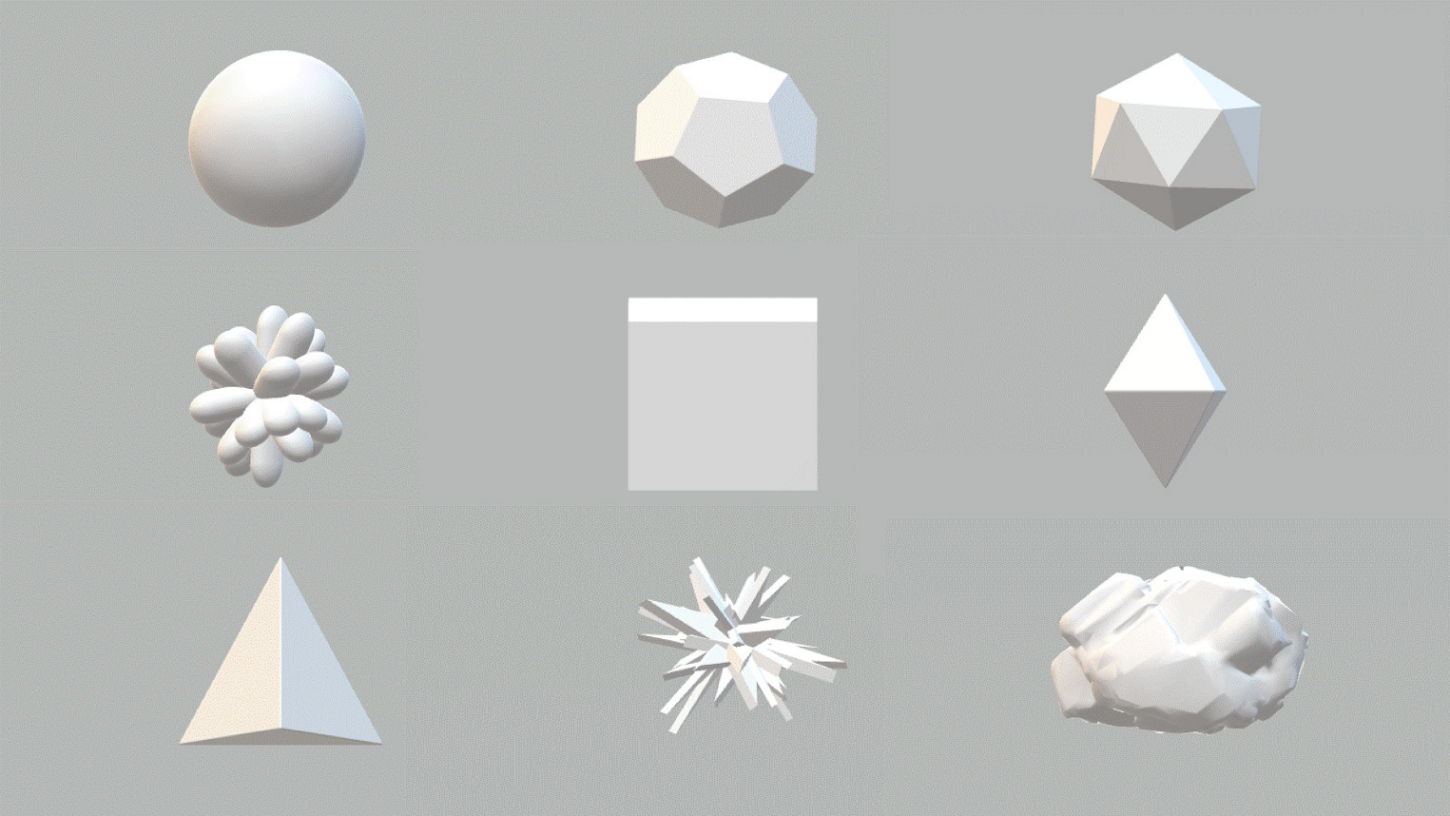
Examples of the experimental stimuli used in Experiment 1. From the upper left corner: Sphere, dodecahedron, icosahedron (upper row), custom-made rounded irregular shape, cube, octahedron (middle row), tetrahedron, custom-made angular irregular shape, and the naturalistic irregular control shape (lower row).

The study was conducted online on the Testable platform (www.testable.org; Rezlescu et al., 2020) between April 18, 2022 and June 18, 2022. The experimental stimuli consisted of nine GIF images representing the different geometric shapes: a sphere, the two custom-made irregular angular and rounded shapes, another naturalistic irregular shape modelled on a natural rock, together with the five Platonic solids (a tetrahedron, an octahedron, an icosahedron, a cube, and a dodecahedron). The stimuli were created with the help of the Blender software (Version 3.2), such that all geometric shapes had the same light grey color and the same height (5.8 cm/5°32′ degrees of visual angle, assuming a standard viewing distance of 60 cm from monitor); the complete image (object + background) presented to the participants each trial was 9.3 cm high × 17.3 cm wide. The turntable animation property was used in Blender, such that all of the geometric shapes rotated continuously in the GIF file. That is, throughout the experimental trial, the 3D geometric shape was perceived in continuous rotating motion. See Figure 2 for static 2D examples of the geometric shapes used in Experiment 1. The experimental stimuli, together with the data that support the findings of this study, are available from the corresponding author, [GJ], upon request.

#### Procedure and Experimental Design

At the beginning of the experiment, the participants were asked to enter into full-screen mode and calibrate their screens, such that all participants saw the experimental stimuli the same size. The experimental platform presented the participants with instructions outlining the general scope of the study and the type of data that would be collected as a result of their taking part. They were informed that they could quit at any point, gave their informed consent, and provided the required demographic data. Each experimental trial consisted of the presentation of a single experimental stimulus (i.e., a rotating geometric shape) together with a visual analog scale (VAS). The participants rated the presented shape on the given VAS, with only the end anchors visible. The VASs used were set from 0 to 100 points, where 0—lack of the measured variable, 50—moderate presence, and 100—maximum recorded. Note that the VAS provides an effective means of capturing characteristics that vary along a continuum which cannot always be measured directly. They also provide an excellent means of capturing participants’ subjective response along a measured scale (see Delgado et al., 2018; Hayes & Patterson, 1921; Yeung & Wong, 2019).

The manipulated independent variable was Geometric shape (naturalistic irregular shape, sphere, angular irregular shape, rounded irregular shape, tetrahedron, octahedron, icosahedron, cube, dodecahedron). Each geometric shape was presented until a response was detected, for each of the three VASs recorded as dependent measures: Liking (very unpleasant – very pleasant), Hardness (very soft – very hard), and Temperature (very cold – very hot). Four repetitions were recorded for each visual stimulus. In total, each participant received 108 trials in randomized order. The experiment lasted for between 15 and 20 min.

#### Data Analysis

The data from each of the three VASs (Liking, Hardness, and Temperature) were inspected visually with histograms and sample/theoretical quantiles (*Q–Q* plots), together with skewness and kurtosis calculations. Following the previous literature (e.g., the Bouba-like/Kiki-like shapes), to investigate the rounded-shape effect in 3D, the Liking scale data were consequently analyzed with a one-way repeated-measures analysis of variance (ANOVA) with the factor Geometric shape (naturalistic irregular shape, sphere, angular irregular shape, or rounded irregular shape). The Liking ANOVA was followed-up with a set of post-hoc hypothesis-driven tests in order to investigate the existence of a *rounded-shape effect*, by comparing the sphere, with all of the irregular control shapes.

The second set of analyses intended to compare the Platonic solids to the sphere. For this, all the VASs were analyzed with one-way repeated-measures ANOVAs with the factor of Geometric shape (naturalistic irregular shape, sphere, tetrahedron, octahedron, icosahedron, cube, dodecahedron). Note that the irregular angular/rounded shapes were not used in these analyses, because no hypothesis existed with respect to the Platonic solids as compared to the Bouba-like/Kiki-like shapes. These ANOVAs were followed-up by paired-samples *t*-tests to identify *any geometric shape effect* in the data, by directly comparing the sphere, the naturalistic control shape, and the five Platonic solids. Greenhouse-Geiser correction was used to adjust the ANOVA degrees of freedom, when the sphericity assumption was violated. The Holm correction was used to account for the familywise-error in the post-hoc paired-sampled *t*-tests (Holm, 1979). Statistical analyses were performed in Matlab (R2021a, MathWorks, Natick, MA, US) and JASP 0.16.1 (JASP Team, 2022).

### Results

**Figure 3.**
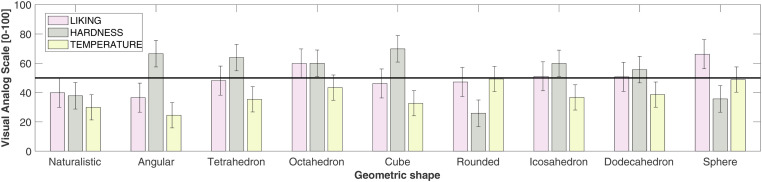
Means ± 95% CI for each of the three visual analog scales (VASs) used to evaluate the geometric shapes in Experiment 1: liking (very unpleasant – very pleasant), hardness (very soft – very hard), and temperature (very cold – very hot). 0 on the VAS scales denotes the very unpleasant (liking), the very soft (hardness), and the very cold (temperature). The thick horizontal line indicates the mid-point of the VAS.

*Liking:* The results indicated a significant main effect of Rounded shape (*F*[3,102] = 7.44; *p *< .001; *η^2^_p_* *=* .180). Specifically following-up the rounded-shape effect, the results indicated that the sphere (*M* = 66.17; *SE* = 4.87) was liked significantly more than both the irregular angular shape (*M* = 36.58; *SE* = 5.55; *t*[34] = −4.31, *p *< .001; *d* = −.925), and the naturalistic irregular shape (*M* = 39.87; *SE* = 5.96; *t*[34] = 3.83, *p* = .001; *d* = .822). Moreover, when comparing all of the geometric shapes, a significant main effect of Geometric shape was evidenced (*F*[3.77,128.28] = 5.11; *p *< .001; *η^2^_p_* *=* .131), with the sphere once again significantly more liked than either the cube (*M* = 46.21; *SE* = 4.99; *t*[34] = −3.65, *p* = .007; *d* = −.686) or the tetrahedron (*M* = 48.14; *SE* = 4.98; *t*[34] = −3.29, *p* = .021; *d* = −.620). From the Platonic solids, the octahedron (*M* = 59.84; *SE* = 4.70) was liked significantly more than the naturalistic irregular control shape (*t*[34] = 3.65, *p* = .007; *d* = .686). None of the other post-hoc *t*-tests survived the familywise-error correction (see Figure 3).

*Hardness*: The results highlighted a significant main effect of Hardness (*F*[2.83,96.47] = 13.61; *p *< .001; *η^2^_p_* *=* .286), indicating that the Platonic solids were rated as looking significantly harder (see Figure 3). The sphere (*M* = 35.66; *SE* = 5.06) was perceived as being significantly softer than the cube (*M* = 69.84; *SE* = 4.03; *t*[34] = 6.84, *p *< .001; *d* = 1.25), the tetrahedron (*M* = 63.93; *SE* = 4.41; *t*[34] = 5.66, *p *< .001; *d* = 1.03), the octahedron (*M* = 59.96; *SE* = 4.59; *t*[34] = 4.86, *p *< .001; *d* = .886), the icosahedron (*M* = 59.89; *SE* = 4.19; *t*[34] = 4.85, *p *< .001; *d* = .883), and the dodecahedron (*M* = 55.64; *SE* = 4.02; *t*[34] = 4.00, *p* = .001; *d* = .729). A very similar pattern of results was also observed for the naturalistic irregular control shape (*M* = 37.77; *SE* = 5.86), which was perceived as being significantly softer than the cube (*t*[34] = 6.42, *p *< .001; *d* = 1.17), the tetrahedron (*t*[34] = 5.23, *p *< .001; *d* = .954), the octahedron (*t*[34] = 4.44, *p *< .001; *d* = .809), the icosahedron (*t*[34] = 4.43, *p *< .001; *d* = .806), and the dodecahedron (*t*[34] = 3.58, *p* = .005; *d* = .652). Importantly, the sphere did not differ significantly in terms of its expected hardness from the naturalistic irregular shape (*p* = n.s.).

*Temperature*: The results of Experiment 1 indicated a significant main effect of Temperature (*F*[3.92,133.36] = 4.33; *p* = .003; *η^2^_p_* *=* .113); see Figure 3. Post-hoc tests highlighted that the sphere (*M* = 48.93; *SE* = 4.30) was rated as significantly warmer than the cube (*M* = 32.67; *SE* = 4.67; *t*[34] = −3.69, *p* = .006; *d* = .628), the tetrahedron (*M* = 35.36; *SE* = 4.43; *t*[34] = −3.08, *p* = .044; *d* = −.525), and the neutral control shape (*M* = 29.87; *SE* = 4.18; *t*[34] = −4.33, *p *< .001; *d* = −.736). In this case, the geometric naturalistic irregular shape was rated as looking significantly colder than the octahedron (*M* = 43.39; *SE* = 4.64; *t*[34] = 3.07, *p* = .044; *d* = −.736).

### Discussion

The results of Experiment 1 confirm that the sphere is the most liked of the geometric shapes. The additional scale results reinforce the notion that the sphere was rated significantly different in terms of its hardness and temperature: The sphere is visually perceived as being softer and also thermally moderately warm. As far as the Platonic solids are concerned, we find significant differences in all three of the recorded measures. The results highlight, quite unexpectedly, that geometric shapes closest to the sphere, such as the custom-built rounded irregular shape, the icosahedron, and the dodecahedron, but also, in certain respects the naturalistic irregular control shape, received similar ratings as far as liking, hardness, and temperature are concerned. This result could be indicative of a general effect of a rounded shape giving rise to increased liking (cf. Lee, 2018). Having acknowledged the rounded shape effect, our results also suggest a clear effect of symmetry, with both the naturalistic and the angular irregular shapes being rated as significantly less pleasant than the regular symmetric geometric shapes.

Temperature assessments are all situated below/on the moderate demarcation of the scale used in our study. Interestingly, the results of Experiment 1 indicate that assessments of temperature for the geometric shapes that were presented are generally aligned with those of liking, thus indicating that rounded shapes are evaluated as warmer, with the angular geometric shapes being thermally evaluated as closer to the cold end of the VAS. Here, it is worth mentioning that certain of the Platonic solids (e.g., the octahedron or the dodecahedron) were evaluated as being very similar to the sphere (i.e., moderately warm), apparently indicating a symmetry effect as far as geometric shape – temperature correspondences are concerned.

Experiment 1 was designed to assess regular versus irregular geometric shapes with respect to pleasantness, together with other sensorial qualities, such as expected hardness and temperature. The geometric shapes were presented in a neutral grey color, so as not to induce any implicit taste–color correspondence; it has been demonstrated that people tend to associate the texture of whitish cotton candy with the taste of sweetness (Spence et al., 2019). In Experiment 2, we chose the preferred Platonic solids from Experiment 1 (namely the octahedron, the icosahedron, and the dodecahedron), together with the sphere, and the control naturalistic irregular shape, and we investigated whether what people like with respect to geometrical shape, is also evaluated as being tastier. For this, each of the chosen shapes was fitted with edible and inedible visual textures (Barbosa Escobar et al., 2022; Slocombe et al., 2016) and the participants rated how tasty they expected them to be, as well as judging their perceived texture. We hypothesized significant enhancement in expected tastiness for those shapes that were preferred. Additionally, we expected to see a boost in tastiness ratings for those textures that reminded people of edible foods.

## Experiment 2

### Methods

#### Participants

An *a priori* power calculation with an *α* = .05 and 1−*β* = .80 indicated that a sample size of 60 participants was necessary to detect an effect of *f* = .25 in the data (G*Power 3.1; Erdfelder et al., 2009; Faul et al., 2007), given that an effect was present (Cohen, 1988). A total of 73 participants took part in Experiment 2 (47 female; mean age of 27 years, SD = 11 years, age range: 18–62 years). The experimental methods are very similar to those of Experiment 1, with the following exceptions.

#### Materials

**Figure 4.**
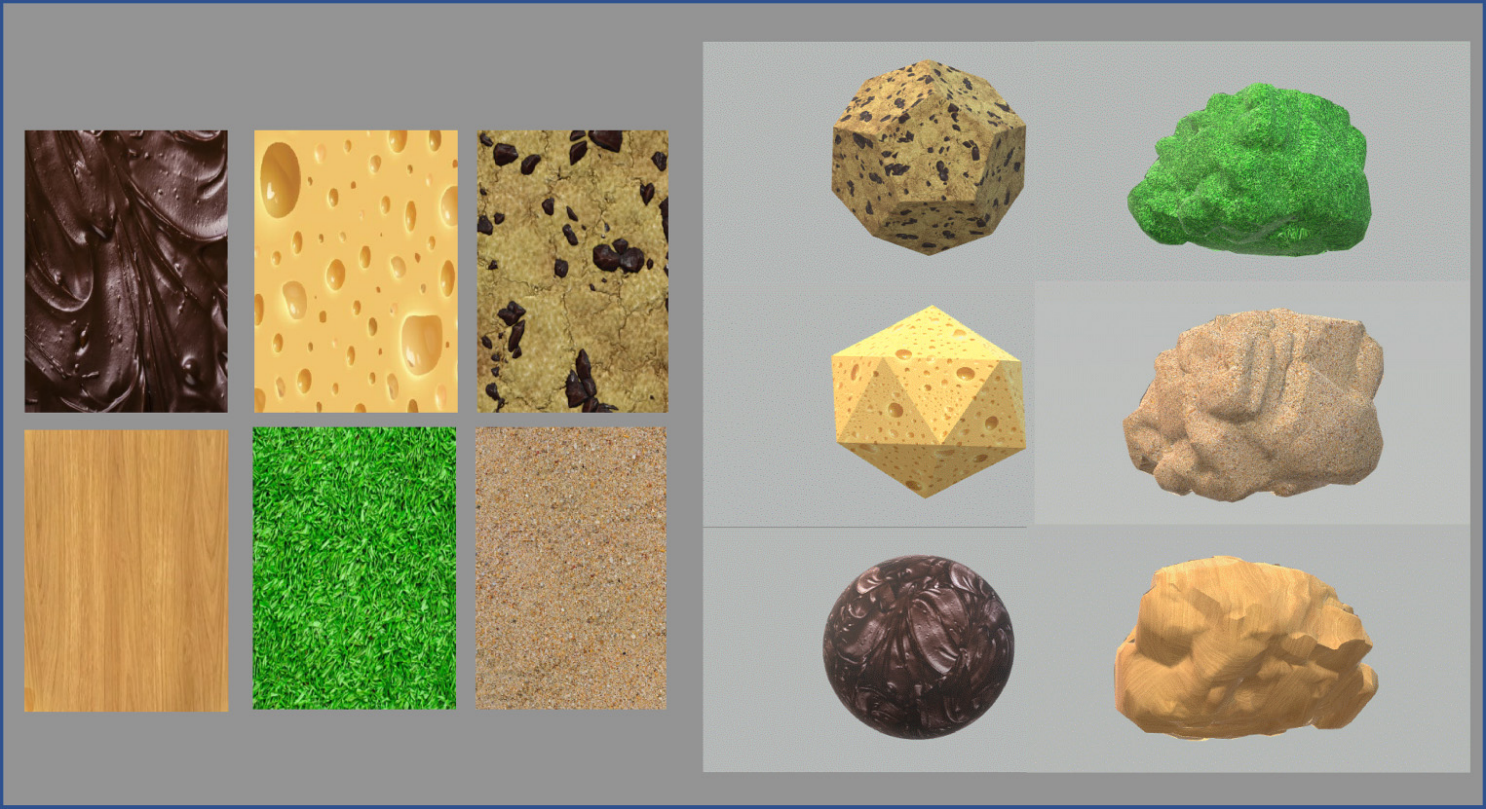
Experimental textures used in Experimental 2 (upper row, left: chocolate, cheese, cookie; lower row, left: wood, grass, sand). Examples of experimental geometric shapes fitted with various textures are presented in the rightmost two columns.

The experimental stimuli consisted of GIF images of the five 3D geometric shapes (sphere, icosahedron, dodecahedron, octahedron, and the naturalistic irregular control shape), each fitted with one of the edible (Swiss cheese, cookie, and chocolate) and inedible textures (grass, sand, and wood). All of the images had the same height (9.3 cm) and width (17.3 cm), with the contained object set to a height of 7 cm/6°40′ degrees of visual angle, when assuming a standard distance of 60 cm from monitor. The 3D stimuli were rotated with the in-built turntable Blender function. Six 2D stimuli depicted the individual textures of the experiment (Swiss cheese, cookie, chocolate, grass, sand, and wood; see Figure 4 for examples of the various textures used).

#### Experimental Design and Procedure

Within the experiment, each combination of texture × geometric shape was repeated twice. The manipulated variables were Geometric shape (sphere, icosahedron, dodecahedron, octahedron, and the naturalistic irregular control) and Texture (Swiss cheese, cookie, chocolate, grass, sand, and wood). The dependent variables were two 0–100 VASs for Tastiness (not at all tasty – very tasty) and Appearance (very smooth – very rough). Each participant completed a total of 120 experimental trials. At the end of the experiment, the six 2D shapes were presented in a randomized order, together with two buttons (Edible and Inedible) and the participants were required to make a choice according to what they thought each texture represented.

#### Data Analysis

Averages of the data collected for the two VASs were analyzed with repeated-measures ANOVAs with the factors Geometric shape (naturalistic irregular control, sphere, icosahedron, dodecahedron, or octahedron) and Texture (cheese, cookie, chocolate, grass, sand, or wood), followed by Holm-corrected *t*-tests.

### Results

*Tastiness*: The results indicated a significant main effect of Geometric shape (*F*[2.86,205.92] = 16.35; *p *< .001; *η^2^_p_* *=* .185) on tastiness ratings. The sphere (*M* = 52.73; *SE* = 1.51) was rated as significantly tastier than the icosahedron (*M* = 46.48; *SE* = 1.69; *t*[72] = −5.27, *p *< .001; *d* = −.245), the dodecahedron (*M* = 47.75; *SE* = 1.51; *t*[72] = −4.20, *p *< .001; *d* = −.195), the octahedron (*M* = 45.62; *SE* = 1.69; *t*[72] = −5.99, *p *< .001; *d* = −.279), and the naturalistic irregular control shape (*M* = 43.71; *SE* = 1.87; *t*[72] = −7.60, *p *< .001; *d* = −.354). The naturalistic control shape looked significantly less tasty than the Platonic dodecahedron (*t*[72] = −3.40, *p* = .005; *d* = −.158).

**Figure 5.**
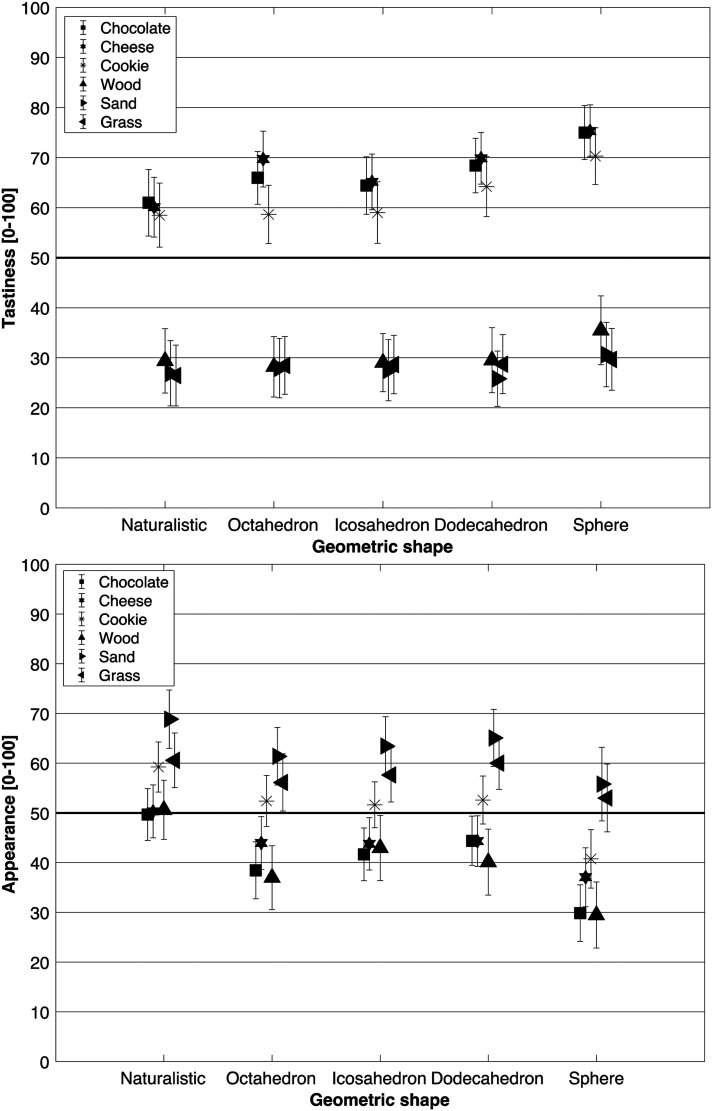
Means ± 95% CI for each of the two visual analog scales (VASs) used to evaluate the geometric shapes in Experiment 2: tastiness (not at all tasty – very tasty, upper graph), appearance (very smooth – very rough, lower graph). 100 on the VAS scales denotes the very tasty (tastiness) and the very rough (appearance). The thick horizontal line indicates the mid-point of the VAS.

A main effect of Texture (*F*[1.72,123.82] = 75.21; *p *< .001; *η^2^_p_* *=* .511) was also evident on tastiness ratings, as well as an interaction between Geometric shape and Texture (*F*[11.79,848.92] = 2.49; *p* = .004; *η^2^_p_* *=* .033; see Figure 5). These effects were primarily driven by the edible textures looking significantly tastier than the inedible textures (all *p*s* *< .001; see Figure 4). Because of the difficulty associated with achieving a balanced experimental design with such diverse edible/inedible textures, we chose to average over all edible/inedible shapes and subsequently compared them with paired-sample *t*-tests. The results indicated that the edible shapes (*M* = 65.69; *SE* = 2.11) were rated as looking significantly tastier than the inedible shapes (*M* = 28.83; *SE* = 2.58; *t*[72] = 9.97, *p *< .001; *d* = 1.17), as expected. None of the hypothesis-driven post-hoc *t*-tests performed following the two-way interaction (e.g., investigating any texture differences between the various Platonic solids) survived the correction for multiple comparisons.

*Appearance*: The results highlighted a significant main effect of Geometric shape (*F*[2.24,160.91] = 15.14; *p *< .001; *η^2^_p_* *=* .174) on the appearance data. The sphere (*M* = 40.99; *SE* = 1.99) was rated as looking significantly smoother than all of the Platonic solids tested in Experiment 2, including the icosahedron (*M* = 50.19; *SE* = 1.41; *t*[72] = 4.50, *p *< .001; *d* = .374), the dodecahedron (*M* = 51.08; *SE* = 1.33; *t*[72] = 4.93, *p *< .001; *d* = .410), the octahedron (*M* = 48.22; *SE* = 1.50; *t*[72] = 3.53, *p* = .003; *d* = .294), and the naturalistic irregular control shape (*M* = 56.55; *SE* = 1.66; *t*[72] = 7.61, *p *< .001; *d* = .633). On the other hand, the naturalistic irregular control shape was rated as looking significantly rougher than the Platonic octahedron (*t*[72] = 4.08, *p *< .001; *d* = .339), the icosahedron (*t*[72] = 3.11, *p* = .010; *d* = .259), and the dodecahedron (*t*[72] = 2.67, *p* = .032; *d* = .222).

There was a significant main effect of Texture (*F*[2.72,195.94] = 18.39; *p *< .001; *η^2^_p_* *=* .203) in the appearance data. Post-hoc tests indicated that the chocolate texture (*M* = 40.81; *SE* = 2.00) was rated as looking significantly smoother than the cookie texture (*M* = 51.33; *SE* = 1.80; *t*[72] = −3.39, *p* = .006; *d* = −.428), sand (*M* = 62.92; *SE* = 2.42; *t*[72] = −7.12, *p *< .001; *d* = −.899), and grass (*M* = 57.48; *SE* = 2.36; *t*[72] = −7.49, *p *< .001; *d* = −.678). Furthermore, the cheese texture (*M* = 43.89; *SE* = 2.14) appeared significantly smoother than the sand (*t*[72] = −6.12, *p *< .001; *d* = −.774), and grass textures (*t*[72] = −4.37, *p *< .001; *d* = −.553). The cookie texture was rated as looking rougher than the wood (*M* = 40.03; *SE* = 2.47; *t*[72] = 3.64, *p* = .003; *d* = .460), but smoother than the sand texture (*t*[72] = −3.73, *p* = .002; *d* = −.471). Lastly, amongst the inedible textures, wood was rated as being significantly smoother than both sand (*t*[72] = −7.37, *p *< .001; *d* = −.931) and grass (*t*[72] = −5.62, *p *< .001; *d* = −.710). Averaging over all edible/inedible shapes’ appearance data, and comparing them with paired-sampled *t*-tests revealed that the edible shapes appeared overall significantly smoother (*M* = 45.34; *SE* = 1.54), than the inedible shapes (*M* = 53.47; *SE* = 1.43; *t*[72] = −3.52, *p *< .001; *d* = −.412).

The results highlighted a significant interaction between Geometric shape and Texture (*F*[13.38,963.14] = 1.86; *p* = .029; *η^2^_p_* *=* .025; see Figure 5). An evident hierarchy can be seen in Figure 5, with sand receiving the roughest evaluations, followed by grass, and then the cookie texture, whereas the regular shapes given textures of wood, chocolate, and Swiss cheese were rated as smoother. However, this pattern of results did not hold for the irregular naturalistic shape, our control geometric shape, modelled from a naturalistic-looking rock. First, as can be seen in Figure 5, for the naturalistic control shape, all appearance ratings are above 50, indicating that when irregular, even the smoothest materials were associated with a rather rougher texture. It is the rounded sphere that exhibits the smoothest ratings. None of the *t*-tests performed following the two-way interaction, to detect any differences in appearance, survived the correction for multiple comparisons for the platonic-shaped objects paired with any of the six textures that were tested.

### Discussion

The results of Experiment 2 highlight that tastiness ratings were influenced significantly by the geometric shape and texture of the visually presented foods, confirming previously reported results concerning geometric shape – taste interactions. As expected, the participants rated the edible textures as looking tastier and smoother, as compared to the inedible textures. From the edible textures, chocolate was smoothest, whereas, from the inedible textures, wood received the smoothest ratings. At the other end of the scale, the edible cookie texture was perceived as rather rough, the same as for the natural texture of sand.

Texture interacted with geometric shape, as manipulated in our second experiment. These results clearly confirm the superior liking for the sphere from Experiment 1. As such, not only is the sphere most liked, but it also looks the tastiest. When paired with edible textures, the spherical presentation of foodstuffs results in enhanced ratings of tastiness. This result accords with studies on expected versus experienced chocolate of diverse angular/rounded shapes – Rounded chocolates are expected to taste sweeter, less bitter, and creamier (e.g., Spence, 2013; Wang et al., 2017b). Importantly, spherical-like shapes (e.g., the icosahedron and dodecahedron from the Platonic solids), which clearly follow the outline of the sphere, are perceived as looking significantly tastier than nonspherical Platonic solids (e.g., the octahedron tested in Experiment 2). For tastiness, a hierarchy is evident, with the sphere at the top, the various Platonic solids in the middle, and the naturalistic irregular control shape at the bottom.

It thus appears that regular and symmetrical geometric shapes are overall tastier than irregular shapes. To take the example of the cookie, when shaped irregularly, it was rated as only moderately tasty, but when rounded, its rating approached the top of the tastiness scale. This result could be linked to the rough mouthfeel characteristics that have been reported when tasting a cookie from a rough plate (Biggs et al., 2016), even though the plate being rough or not seems to not always make a difference in taste assessments, but nevertheless rough textures do tend to be evaluated as sour (Slocombe et al., 2016), which, in turn, is a good candidate taste for asymmetrical geometrical shapes (Deroy & Valentin, 2011). In this context, the shape of the regular Platonic solids could act as an (until-now) hidden moderator of the texture-to-taste crossmodal correspondence. Note that Swiss cheese was rated as appearing smoother when presented in a spherical form, suggesting that the expectation of a certain taste could have blended amongst the presentation of angular/rounded shapes (Gal et al., 2007). Other studies have focused on the shape of the transparent windows typically used in the design of various commercial packaging, finding the expected sweet-round crossmodal pairing to result in tastier evaluations, with mixed results for the more complex angular/rounded shapes (Simmonds et al., 2019). Future studies need to assess whether the expected liking and tastiness of the perfectly symmetrical Platonic solids accords with the perceived oral-somatosensory appreciation of actual food bearing such geometric shape.

To further our discussion of shape and taste, the basic tastes associated with the Platonic solids first need to be defined. An extensive literature on rounded shapes has typically paired rounded shapes with sweet tastes (Cytowic & Wood, 1982; Spence & Deroy, 2014), though, at present, the basic taste matching with each of the Platonic solids has not been studied. In Experiment 3, the participants had to assess the corresponding basic taste for each of the five Platonic solids, together with the sphere, the control naturalistic irregular shape, as well as the two custom-built irregularly angular/rounded shapes; that is, all of the geometric shapes used previously in Experiments 1 and/or 2. The expectation was that rounded shapes would be evaluated as looking sweeter, whereas angular shapes would attract more sour ratings (Deroy & Valentin, 2011).

## Experiment 3

### Methods

An a priori power calculation with an *α* = .05 and 1–*β* = .80 indicated that a sample size of 207 participants was necessary to detect an effect size of *w* = .25, in the data (G*Power 3.1; Erdfelder et al., 2009; Faul et al., 2007), given that an effect was present (Cohen, 1988). A total of 254 participants took part in Experiment 3. We set the age limit for inclusion in the study at 16 years of age. We also excluded those participants with zero variance in their responses (i.e., they always pressed the same button, for all nine of the geometric shapes tested in Experiment 3), as they showed a lack of engagement with the experimental task, together with an outlier exclusion in the RT data (i.e., answers consisting of a single button being pressed, or RTs that were too fast being recorded, e.g., RT*_z_*_-score_ > 3, indicating that the same button was pressed, without the participant truly considering the presented geometric shape). The remaining sample (*N* = 210, 143 female) had a mean age of 26 years old (*SD* = 11 years, age range: 16 – 60 years).

The apparatus and materials were the same as in Experiment 1. The participants were informed that the experiment was designed to investigate any links between basic tastes and geometric shapes, were given a short introduction to the fifth basic taste – umami, for anyone who was unfamiliar with it (Ikeda, 2002), after which they gave their consent to take part. The participants received nine trials, each with a single 3D rotating geometric shape, and five taste buttons, placed below the shape: Sour, Bitter, Sweet, Salty, and Umami. On each trial, participants were instructed to choose the taste that they thought was most representative for the currently displayed geometric shape, by clicking on one of the five available buttons. They were informed that there were no correct or incorrect answers, we required their subjective opinion on the shape – taste match. Once their choice had been made, the experiment continued on to the next trial. The order of the trials was randomized across participants. The experiment took 1–2 min to complete.

For each of the geometric shapes, we calculated frequency tables of the five basic tastes and we then used Pearson's Chi-squared test to compare the observed frequencies in our sample to the taste frequencies predicted by chance. For the analysis, we considered equal predicted frequencies for each of the basic tastes in each of the nine geometric shapes tested in Experiment 1 (i.e., each basic taste with a predicted frequency of 20%).

### Results

**Figure 6.**
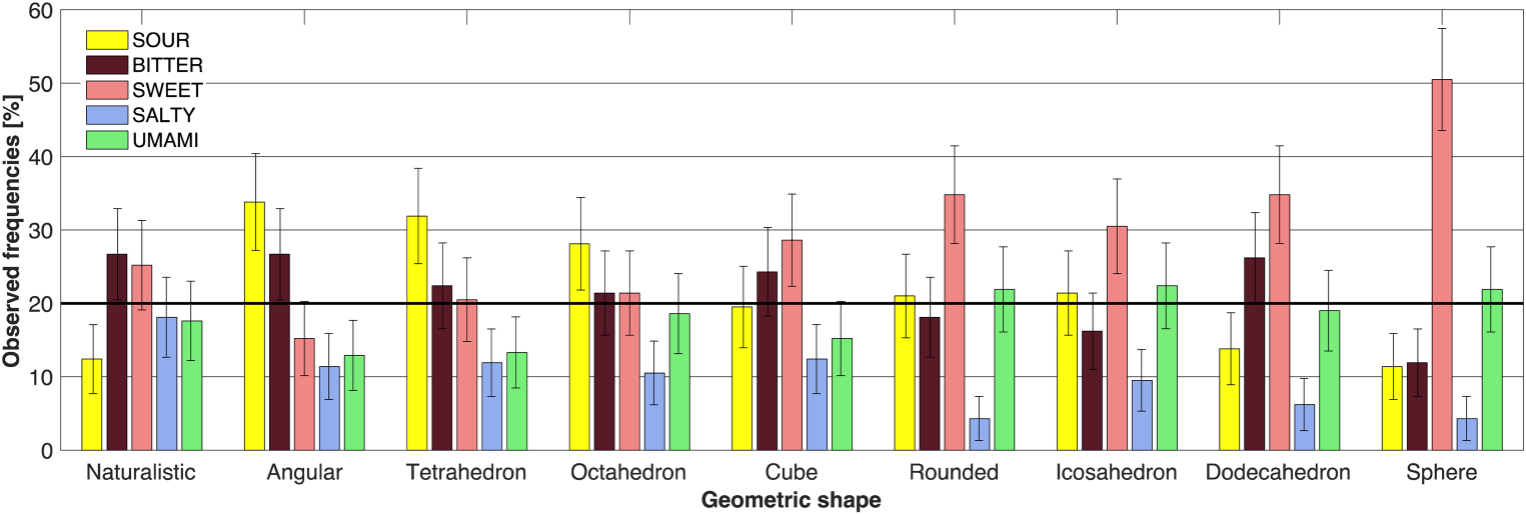
Means ± 95% CI (presented as percentages) for each of the five basic tastes assessed to represent each of the nine geometric shapes in Experiment 3. The participants were asked to pair one of the five basic tastes to each geometric shape presented. The thick horizontal line indicates chance-level responding (20%).

Figure 6 outlines the basic taste frequency data (means ± 95% CI) in Experiment 3, for each of the nine geometric shapes. The shapes are ordered on the *x*-axis from the naturalistic irregular shape on the left, through the rather angular shapes of the irregular angular shape and Platonic tetrahedron, octahedron, and the cube, up to the rounded shapes of the irregular rounded shape, the Platonic icosahedron and dodecahedron, with the sphere positioned furthermost on the right of Figure 6. Visual inspection of the figure makes clear that some of the tastes have not surpassed chance (depicted by the thick black line, at 20%), with certain of the geometric shapes exhibiting a strong effect of a rather obvious single above-chance chosen taste (e.g., see the sweet taste of the sphere; χ^2^[4] = 138.43; *p *< .001), some exhibiting two tastes chosen at above-chance levels (e.g., the naturalistic irregular shape, χ^2^[4] = 14.62; *p* = .006; irregular angular shape, χ^2^[4] = 40.14; *p *< .001; cube, χ^2^[4] = 18.14; *p* = .001; dodecahedron, χ^2^[4] = 51.05; *p *< .001), or even three (the tetrahedron, χ^2^[4] = 27.05; *p *< .001; the octahedron, χ^2^[4] = 17.05; *p* = .002; irregular rounded shape, χ^2^[4] = 49.67; *p *< .001; the icosahedron, χ^2^[4] = 25.38; *p *< .001). Another visually striking pattern in the data is the change in associated taste, evident through the clustering of sour and bitter frequencies for the angular shapes depicted on the left of Figure 6, and the overall sweet and umami frequencies found for the rounded geometric shapes grouped on the right side of Figure 6. It is interesting to consider the cube that is one of the Platonic solids depicted in the middle, which was rated as looking both significantly bitter and significantly sweet at the same time.

### Discussion

The results of Experiment 3 demonstrate that our participants tended to pair the rounded shapes with the tastes of sweet and umami, whereas the more irregular and angular shapes were associated with the basic tastes of sourness and bitterness instead. It should be noted that these results are in line with classic studies on shape-taste associations (Cytowic & Wood, 1982; Spence & Deroy, 2014; see also Huisman et al., 2016) as well as more recent studies on the correspondence in people on the autistic spectrum (Chen et al., 2021). Interestingly, the salty basic taste failed to surpass chance level for geometric shape–taste pairing in Experiment 3. The salty taste nevertheless received the highest frequency of responses when paired with the control irregular shape that was used in the experiments presented in this study. Sour was dominant amongst the angular and irregular geometric shapes tested in Experiment 3. In the General Discussion, we consider several points for explanation and a few suggestions for potential application.

## General Discussion

We present the results of three online studies designed to investigate symmetry in judgments of liking for various rounded as opposed to angular shapes. To this end, our main interest was in evaluating the perfectly symmetrical Platonic solids, a set of five regular polyhedra first described by Plato. These are regular shapes that occur in nature and because they are perfectly symmetrical, in a rotational sense, it has been suggested that their structure is derived from symmetry and in turn, reflects beauty (Wilczek, 2015). Because of the well-known effect of enhanced liking for rounded/spherical shapes, we chose a sphere as one of our 3D stimuli, as well as custom-designing another two irregular, both rounded and angular, 3D shapes, together with one other custom-made irregular naturalistic-looking 3D shape. In line with the results that have been reported previously, our findings indicate a clear preference for rounded shapes, with moderate liking being expressed for the regular symmetrical, but nevertheless still angular, Platonic solids. The most liked shapes are also considered the tastiest and, as expected, pairing edible textures with regular shapes results in enhanced judgments of tastiness. Experiment 3 establishes the intuitive geometric shape–taste pairing, for all of the shapes used in this study.

The sphere was liked best of all and also, along with our custom-made irregular rounded shape, was rated as appearing warmest and softest, amongst all of the geometric shapes tested in Experiment 1. It has been demonstrated that context-free geometric shapes can act as emotional primitives and thus they can convey emotion, with pointy downward-facing V's perceived as threatening, as opposed to their rounded counterparts, which are rated as pleasant (Larson et al., 2009; Larson et al., 2012). The same angular/rounded demarcation would appear to be present, context-free, in the affective domain. It has been demonstrated that people do, in fact, associate rounded shapes with rather positive emotion across sensory systems and higher-level concepts (Blazhenkova & Kumar, 2018) and this affective response is adaptive (Leder et al., 2011). Moreover, if we consider the control custom-made rounded shape, which was purposefully constructed so as to capture the essence of the “bouba-shape” (Spence & Deroy, 2014), this was significantly liked, and rated as both smooth and warm and with a rather sensual appearance (e.g., resembling multiple breasts, see the depiction of goddess Artemis of Ephesus, for an ancient real-world example). Relatedly, the sweet taste has been characterized as being “the smooth of taste,”^1^ so it is unsurprising that our control rounded shape was also significantly paired with the sweet taste.

The Platonic solids, on the other hand, were rated as looking significantly harder and were also associated with a lower temperature. Hardness is a clear characteristic of Platonic solids, as they have all received above-moderate ratings. The hardness of the Platonic shapes is not necessarily anchored in symmetry, but rather, seems to act as a counter to the rounded shape. Angular in our study thus relates to a cooler temperature, hardness, and also to roughness of texture. To follow the Platonic shapes’ implied beauty, the Gestalt principles of harmony, contrast, and dissonance have been put forward as drivers of flavor perception (Hartshorne, 1934, p. 205; see also Spence, 2020a), all these principles applying to the five perfectly symmetrical Platonic solids assessed here.

Because the results of the three experiments presented in this study are based on expected evaluations of liking, hardness, temperature, tastiness, and appearance/texture, when interpreting the present results the distinction between evaluating the *expected* liking or taste, as opposed to actually experiencing the taste, hardness, or temperature of a certain geometric shape, such as this would happen when sampling foodstuffs in the real-world needs to be acknowledged (e.g., see the crossmodal correspondence elicited between sound and temperature, by both imagining and perceiving water of different temperatures, Wang & Spence, 2017). Note that round-shaped foodstuffs (e.g., chocolate) tasted in the 3D form in the real world are rated as creamier than their angular presentation (Baptista et al., 2022; see also Lenfant et al., 2013; Spence, 2014; Wang et al., 2017b). Rounded shapes increase the desirability of hedonic, but not that of utilitarian, foods (Zhou et al., 2021). Interestingly, it appears that round-shaped food is evaluated as having fewer calories than angular/squared-shaped food, with weight as a significant mediator of this shape-to-calories effect (Li et al., 2022). Furthermore, perceived sweetness can be enhanced semantically (e.g., through figurative images of sweet foodstuffs) and also crossmodally, by the presence of sweet odors (van Beilen et al., 2011). To the best of our knowledge, 3D presentations of the diverse Platonic solids have yet to be tested.

Of note, the results of Experiment 3 indicate that some shapes, especially amongst the regular Platonic solids, are not strongly associated with a particular basic taste. For the cube, which was rated as looking hardest, because of its regularity and angularity, its pairing with the bitter taste was expected. Quite unexpectedly, the cube is also paired with the sweet taste. In this match, perhaps dominant would be the learned association, both semantic, as well as visual, with a sugar cube. By contrast, the rounded shapes were expected to set a significant expectation of sweetness, and this was indeed confirmed. However, it is evident from the results of Experiment 3 that rounded symmetrical shapes also correspond to the taste of umami. The color-taste correspondence has been studied intensely, nevertheless, the umami taste hasn’t been, until now, consistently matched with a certain shape, even though it seems that when designers create shapes/colors for specific tastes, they are indeed likely to use a rounded shape (Spence et al., 2015).

Such a taste-versatility as found for the Platonic solids begs the question of how this can be used crossmodally? Could we perhaps top the rounded shape dominance in aesthetic judgments so long as we use hardness, temperature, and/or texture more friendly or fitted to a Platonic solid? It would certainly be interesting to pair the shape of a Platonic solid with another sensorial attribute to elicit a specific desired effect, in the culinary field, but also in design and other creative artistic domains (e.g., see Spence, 2013; Wang et al., 2017b). Such an avenue would open the easily discriminable Platonic solids to be tested within the context of semantic discriminability theory (Mukherjee et al., 2022; Schloss et al., 2018). It would certainly be easy to imagine the beautiful perfectly symmetrical Platonic solids becoming “statement-shapes” for the rightfully-matched concept(s), eventually matched to objects that are both hard in terms of consistency and cooler in terms of temperature, as suggested by the results of the present study. That is, with the current results in mind, how would your ideal Popsicle look like, rounded or rather, taking the shape of a beautiful 3D Platonic solid (see, e.g., Callahan, 2014)? Shape is crucial in what regards innovation in the culinary field (Berkowitz, 1987). Think that even though still exploratory in many respects, 3D printing of food is possible and it is already used both in the hospitality field, and with dedication and right tools, can also be done at home (Mantihal et al., 2020). With the ever-developing technology, personalized and health-optimized 3D-printed foods (Lewis, 2012) that align the well-known taste to (various) sensory qualities correspondences, including the shape – taste correspondence described in the present study, appear to be reasonable culinary choices in the near future.

To consider the implications of the research presented in this manuscript, recent years have seen growing interest in the putative mechanisms underlying the crossmodal correspondences, and their relation with other kinds of crossmodal mapping, such as semantic correspondences and amodal relations (Spence & di Stefano, 2022, submitted). Under those conditions where people are able to bring to mind a particular source object, such as being reminded of the fruit when smelling a strawberry aroma, then the correspondence with the color red would appear linked to the source object (i.e., a semantic mapping). However, when no particular source object comes to mind (e.g., as in the case of the basic tastes which tend to be associated with many different foods), correspondences are more likely to be based on the internalization of the statistical regularities of the environment instead (such as the fact that the majority of fruits tend to transition from green, unripe, and sour through redder, riper, and sweeter). However, in that absence of (or perhaps sometimes in competition with) such statistical correspondences, crossmodal matches are sometimes emotionally-mediated as, for example, when arousing colors and sounds are matched with the arousing taste of chili (see Wang et al., 2017a; see also Foroni et al., 2016). While some researchers have suggested that perceptual similarity might also underpin at least certain crossmodal correspondences (see Spence & Di Stefano, submitted), the evidence supporting such a claim is rare outside the special case of those olfactory stimuli that take on the taste properties in the foods in which they are co-presented (e.g., as in the case of the odor of vanilla being described as sweet, Spence, in press; submitted).

Taken together, our results confirm the dominance of the rounded shape as far as liking is concerned. The liked rounded shapes, together with the Platonic solids tested carry a taste for beauty, as not only are they most liked, but also evaluated as tastiest. Our investigation of rotational symmetry over three experiments further extends the current knowledge, by highlighting that enhanced liking of rounded shapes is supported by thermally warmer evaluations. Importantly, angular symmetrical shapes are perceived as harder and colder in terms of their expected temperature. Future research needs to capitalize on these novel sensorial qualities that define symmetrical angular geometric shapes—dynamically assessed in rotational motion as in the present study—while at the same time considering the potential offered by the symmetrical geometrical shapes taste versatility.

## Notes

1. The Enlightenment philosopher Burke uses breast milk as an example of how “water, oil, and a sort of sweet salt, called the sugar of milk” combine to give a “great smoothness of the taste” (Burke, 2009, p. 130).
